# The *Ercc1* ^−/Δ^ mouse model of XFE progeroid syndrome undergoes accelerated retinal degeneration

**DOI:** 10.1111/acel.14419

**Published:** 2024-11-27

**Authors:** Akilavalli Narasimhan, Seok Hong Min, Laura L. Johnson, Heidi Roehrich, William Cho, Tracy K. Her, Caeden Windschitl, Ryan D. O'Kelly, Luise Angelini, Matthew J. Yousefzadeh, Linda K. McLoon, William W. Hauswirth, Paul D. Robbins, Dorota Skowronska‐Krawczyk, Laura J. Niedernhofer

**Affiliations:** ^1^ Institute on the Biology of Aging and Metabolism University of Minnesota Medical School Minneapolis Minnesota USA; ^2^ Department of Biochemistry, Molecular Biology and Biophysics University of Minnesota Minneapolis Minnesota USA; ^3^ Department of Ophthalmology University of Florida Gainesville Florida USA; ^4^ Department of Ophthalmology and Visual Neurosciences University of Minnesota Minneapolis Minnesota USA; ^5^ Department of Physiology and Biophysics, Department of Ophthalmology, Center for Translational Vision Research University of California Irvine, School of Medicine Irvine California USA; ^6^ Department of Integrative Biology and Physiology University of Minnesota Minneapolis Minnesota USA; ^7^ Present address: Department of Medicine Columbia University Medical Center New York New York USA

**Keywords:** age‐related retinal degeneration, cellular senescence, DNA damage

## Abstract

Age‐related macular degeneration (AMD) is a major cause of vision loss in older adults. AMD is caused by degeneration in the macula of the retina. The retina is the highest oxygen consuming tissue in our body and is prone to oxidative damage. DNA damage is one hallmark of aging implicated in loss of organ function. Genome instability has been associated with several disorders that result in premature vision loss. We hypothesized that endogenous DNA damage plays a causal role in age‐related retinal changes. To address this, we used a genetic model of systemic depletion of expression of the DNA repair enzyme ERCC1‐XPF. The neural retina and retinal pigment epithelium (RPE) from *Ercc1* ^*−/Δ*^ mice, which models a human progeroid syndrome, were compared to age‐matched wild‐type (WT) and old WT mice. By 3‐months‐of age, *Ercc1* ^*−/Δ*^ mice presented abnormal optokinetic and electroretinogram responses consistent with photoreceptor dysfunction and visual impairment. *Ercc1* ^*−/Δ*^ mice shared many ocular characteristics with old WT mice including morphological changes, elevated DNA damage markers (γ‐H2AX and 53BP1), and increased cellular senescence in the neural retinal and RPE, as well as pathological angiogenesis. The RPE is essential for the metabolic health of photoreceptors. The RPE from *Ercc1* ^*−/Δ*^ mice displayed mitochondrial dysfunction causing a compensatory glycolytic shift, a characteristic feature of aging RPE. Hence, our study suggests spontaneous endogenous DNA damage promotes the hallmarks of age‐related retinal degeneration.

## Graphical abstract

Spontaneous endogenous DNA damage drives key hallmarks of age‐related retinal degeneration, including visual impairment, photoreceptor cell loss, dysmorphic RPE, mitochondrial dysfunction, and cellular senescence. Therefore, *Ercc1^−/Δ^* mice represent a valuable model for further mechanistic studies on age‐related retinal degeneration and for rapid testing of potential therapeutic interventions.

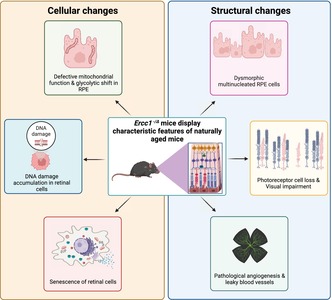

**Abbreviations**
AMD: Age‐related macular degeneration
C12FDG: 5‐Dodecanoylaminofluorescein di‐β‐D‐Galactopyranoside
dB: decibel
Ercc1: Excision repair cross‐complementing group 1
ERG: Electroretinogram
GCL: Ganglion cell layer
H&E: hematoxylin and eosin
INL: Inner nuclear layer
NER: Nucleotide Excision Repair
OCR: Oxygen consumption rate
OKR: Optokinetic response
ONL: Outer nuclear layer
PFA: paraformaldehyde
RPE: Retinal pigment epithelium
RT: room temperature
SASP: Senescence‐associated secretory phenotype
SA‐β‐gal: Senescence‐Associated beta‐galactosidase

## 1 INTRODUCTION

Age‐related macular degeneration (AMD) afflicts 200,000 Americans each year. With our aging population, the number of individuals with AMD will double by 2050, impacting over 5 million people (Varma et al., 2016). AMD is the leading cause of blindness in people over the age of fifty. There are two forms of AMD, a wet or neovascular type, and dry, for which no treatments are available. Therefore, understanding the basic pathogenic mechanisms is needed to develop novel therapeutic approaches.

The retina is the light sensitive neural tissue at the back of the eye and external to the retina is a layer of cells called the retinal pigment epithelium (RPE). The neural retina is composed of three main neuronal layers, and one neuronal type, the ganglion cell, forms the optic nerve that brings visual information into the brain. The RPE is a monolayer of cells connected by tight junctions and forms the outer blood retinal barrier. The apical membrane of RPE faces the photoreceptors and the basolateral membrane of the RPE faces Bruch's membrane. The symbiotic relationship between neural retina and RPE is essential for support of each other's function and any disruption in this regulation leads to vision loss (Sinha et al., 2020). The RPE transports metabolic waste products from the photoreceptors to the blood for removal from the sub‐retinal space and transports nutrients from the blood to the photoreceptors (Strauss, 2005). The retina is highly prone to oxidative stress since it is exposed to light and reactive oxygen species (ROS) generated by the visual transduction pathways (Nishimura et al., 2017). In fact, retinal function is threatened by most of the hallmarks of aging (Kennedy et al., 2014), and our lack of knowledge about the primary drivers of AMD has impeded development of disease arresting or curative therapies.

The hallmarks of aging are biologically interconnected (López‐Otín et al., 2023), meaning each hallmark can be improved by therapeutically targeting one, but also making it highly challenging to define primary drivers of age‐related diseases like AMD. Herein, we test the hypothesis that macromolecular damage, specifically to the nuclear genome, is a primary driver of retinal degeneration. This is based on the heightened levels of oxidative stress in the retina (Hyttinen et al., 2017; Miranda & Romero, 2019) the fact that oxidative DNA damage accumulates with age (Niedernhofer et al., 2018; Wang et al., 2012), an association between reduced expression of several DNA repair genes and AMD (Blasiak et al., 2013; Strunnikova et al., 2005; Synowiec et al., 2012), and the loss of vision seen in genome instability disorders (Liu et al., 2023).

Excision repair cross‐complementing group 1 (*Ercc1*) encodes the non‐catalytic subunit of the ERCC1‐XPF endonuclease. The enzyme is involved in multiple DNA repair pathways including nucleotide excision repair (NER, transcription‐coupled NER and global genome NER) of helix‐distorting DNA adducts (Spivak, 2016), which repairs cyclopurine adducts caused by ROS (Kuraoka et al., 2000), the repair of DNA interstrand crosslinks (Bhagwat et al., 2009), and repair of some double‐strand breaks (Ahmad et al., 2008). In humans, deficiency of ERCC1 or XPF results in various conditions including xeroderma pigmentosum, Cockayne syndrome, cerebro‐oculo‐facio‐skeletal syndrome, Fanconi anemia, and XFE progeroid syndrome (Manandhar et al., 2015; Niedernhofer et al., 2006; Sijbers et al., 1996). These diseases are associated with accelerated aging of multiple organ systems and often visual impairment (Woźniak et al., 2014). Xeroderma pigmentosum patients are particularly affected with eyelid skin cancers, cataracts at young age, retinal thinning, and retinal dystrophy (Dollfus et al., 2003). To test our hypothesis that spontaneous, endogenous DNA damage is a primary driver of AMD, *Ercc1* ^*−/Δ*^ mice were used. These mice model the human XFE progeroid syndrome (Niedernhofer et al., 2006), accumulate oxidative DNA damage more rapidly in several tissues compared to wild‐type mice (WT) (Wang et al., 2012), and age six times faster than WT mice (Yousefzadeh et al., 2020). Retinal tissues from these mice were systematically compared to those of age‐matched WT and old WT mice. By 3–4 months‐of‐age, the *Ercc1* ^*−/Δ*^ mice spontaneously developed many features of retinal aging as seen in old WT mice and associated with AMD, driven by impaired DNA repair. Our data supports the conclusion that endogenous DNA damage can instigate retinal degeneration. The *Ercc1* ^*−/Δ*^ mice may prove to be a model for rapidly testing novel approaches to prevent or treat AMD, including senotherapies (Zhang et al., 2022).

## 2 MATERIALS AND METHODS

### 2.1 Animal model

All experiments were approved by the Institutional Animal Care and Use Committee at the University of Minnesota. *Ercc1* ^*−/Δ*^ mice were bred as previously described (Ahmad et al., 2008). Two‐months to 4‐month‐old *Ercc1* ^*−/Δ*^ animals were selected for this study based on prior evidence of increased senescence in a variety of tissues of mutant animals at this age, which matched the pattern of senescent cell distribution in tissues seen with aging in WT mice (Yousefzadeh et al., 2020), and compared directly with their age‐matched WT and naturally aged WT mice (30‐month‐old). Animals were housed in a light‐and temperature‐controlled facility (lights on from 7 AM to 7 PM, 21°C) with ad libitum access to water and food. All mice studied were in an f1 genetic background established through breeding of an inbred C57BL/6J and an inbred FVB/N mouse.

### 2.2 Optokinetic response

Optokinetic response (OKR) responses were measured using a custom‐built device (ISCAN, Woburn, MA, USA) as described previously (Johnson et al., 2021). After making a sterile incision through the scalp, small screws were put into the crania, and headposts were implanted on the top of the head with Geristore dental cement (DenMat Holdings LLC). Two weeks after headpost surgery, eye movements were recorded. The mice were immobilized by attaching a clamp to the headposts and by placing a tube around their bodies. A video camera is focused on the pupil for recording the eye movements. During testing, the mice were surrounded by a white circular drum with black bars projected onto the white background. The black bars moved left to right across the mouse's visual field with varying spatial frequencies and varying contrasts, and eye movements as they tracked these rotating bars were recorded using a video camera and the ISCAN program. To analyze the data from the ISCAN program, raw files of horizontal and vertical movement components were uploaded into a custom program in R (R Foundation for Statistical Computing). The graphs for each horizontal trace were centered at zero using this average, and the vertical trace was centered at −20 for ease of graphic representation (Johnson et al., 2021). A total of three mice per genotype were examined.

### 2.3 Electroretinogram

Full‐field scotopic and photopic electroretinography (ERG) were conducted on *Ercc1* ^*−/Δ*^ and WT controls using a UTAS Visual Diagnostic System with a Big Shot Ganzfeld dome (LKC Technologies, Gaithersburg, MD, USA), as previously described (Deng et al., 2019). Briefly, mice were dark adapted for 4 h and then anesthetized by an i.p. injection of ketamine (72 mg/kg)/xylazine (4 mg/kg), and scotopic responses were elicited using a series of white flashes of three increasing intensities (−20, −10, and 0 dB). Interstimulus intervals were 2.5, 5.0, and 20.0 s, respectively. Mice were then light adapted to a 30‐cd·s/m^2^ white background for 2 min and photopic responses were elicited, with intensities ranging from −3 to 10 dB (Ye et al., 2016). A‐wave amplitudes and b‐wave amplitudes were calculated as described by Benchorin et al., (2017). A total of three mice per genotype were examined.

### 2.4 RNA isolation and qPCR

Neural retina and RPE/choroid were dissected out from euthanized animals and stored at −80°C. Two eyes from each animal were pooled. Tissues were homogenized by Bullet Blender Tissue Homogenizer (Next Advance), and RNA was isolated using PureLink RNA mini kit (Invitrogen) according to the manufacturer's instructions. Total RNA was quantified using a NanoDrop (ThermoFisher). 1500 ng of total RNA from neural retina and 500 ng of total RNA from RPE/choroid were used to generate cDNA using a High‐Capacity cDNA reverse transcription kit (Applied Biosystems) according to the manufacturer's instructions. Gene expression changes were quantified by qRT‐PCR using 10 μL reaction volume in a QuantStudio Real‐time PCR system (Applied Biosystems) using SYBR Green PCR master mix. For each sample, reactions were performed in triplicate (*n* = 4 biological replicates per group). Data were analyzed by the ΔΔCt method, and the gene expression was normalized to *Gapdh*. The primer sequences are listed in Table 1.

**TABLE 1:** List of primers.

Primer	Sequence
*Gapdh*	Fwd 5′—AAGGTCATCCCAGAGCTGAA—3′
	Rev 5′—CTGCTTCACCACCTTCTTGA—3′
*p16* ^*Ink4a*^	Fwd 5′—CCCAACGCCCCGAACT—3′
	Rev 5′—GCAGAAGAGCTGCTACGTGAA—3′
*p21* ^*Cip1*^	Fwd 5′—TTGCCAGCAGAATAAAAGGTG—3′
	Rev 5′—TTTGCTCCTGTGCGGAAC—3′
*Tnf*	Fwd 5′—ATGAGAAGTTCCCAAATGGC—3′
	Rev 5′—CTCCACTTGGTGGTTTGCTA—3′
*Il6*	Fwd 5′—CTCTGGGAAATCGTGGAAAT—3′
	Rev 5′—CCAGTTTGGTAGCATCCATC—3′
*Mcp1*	Fwd 5′—GCATCCACGTGTTGGCTCA—3′
	Rev 5′—CTCCAGCCTACTCATTGGGATCA—3′
*Pai1*	Fwd 5′—GACACCCTCAGCATGTTCATC—3′
	Rev 5′—AGGGTTGCACTAAACATGTCAG—3′
*Glut1*	Fwd 5′—GCATAGTTACAGCGCGTC—3′
	Rev 5′—CGAACTGCAGTGATCCGAG—3′
*Hk*	Fwd 5′—TGTGAGATTGGACTCATCGTG—3′
	Rev 5′—CCACCATCTCCACGTTTTTC—3′
*Pedf*	Fwd 5′—TGGCTTACTTCAAGGGGCAG—3′
	Rev 5′—CATCATGGGGACTCTCACGG—3′
*Vegfa*	Fwd 5′—TGGGTGCTAAATGGCAGGAG—3′
	Rev 5′—TGTTCTGTCTTTCTTTGGTCTGC—3′
*Kdr*	Fwd 5′—TCCATGTGATCAGGGGTCCT—3′
	Rev 5′—GCACAACAGGGACACACTCT—3′

### 2.5 Histology (H&E staining)

For histological examination, mouse eyes were fixed in Davidson's fixative, paraffin embedded, sectioned at (6 μm), and stained with hematoxylin and eosin (H&E). Images of H&E‐stained retinas were acquired (Huron TissueScope LE). The retinal thickness, outer nuclear layer (ONL) thickness, and photoreceptor cell counts were measured at 6 consecutive distances from the optic nerve head (ON) using ImageJ software. Retinal sections (*n* = 3 retinal sections per eye) from *n* = 4 mice for each genotype were analyzed.

### 2.6 Color fundus photography

Color fundus photography was conducted on *Ercc1* ^*−/Δ*^ and WT controls to comprehend retinal alterations, including retinal thinning, the presence of lesions, and retinal depositions. Mice were anesthetized with an intraperitoneal injection of ketamine (50 mg/kg) and xylazine (10 mg/kg) and subjected to pupillary dilation with topical eye drops containing 0.5% tropicamide and 0.5% phenylephrine. Color fundus photography was performed with a color fundus camera (Phoenix MICRON® 5). A total of six mice per genotype were examined.

### 2.7 Fluorescent immunostaining

To compare the distribution of 53BP1 and γ‐H2Ax in young and old WT and *Ercc1* ^*−/Δ*^ mouse eyes, the tissue was enucleated and immediately fixed in 10% buffered formalin for 48 h. After paraffin infiltration, retinal sections of 6 μm thickness were taken central, through the optic nerve. Prior to antibody staining, sections were deparaffinized and underwent antigen retrieval by heating in 10 mM sodium citrate, 0.05% Tween20 (pH 6.0) for 24 min at 92°C. After washing the slides, non‐specific immunoglobulin binding was blocked for 30 min with 10% normal donkey serum. The cross sections were immunostained overnight for the presence of 53BP1, 1:1000 (Novus, Centennial, CO) and γ‐H2Ax, 1:100 (Abcam, Cambridge, MA). The reaction product was visualized using secondary antibodies Alexa Fluor 594 and Alexa Fluor 488 (Molecular Probes Inc., Eugene, OR), incubated for 3 h at room temperature. After a final wash, the slides were cover slipped with Vectashield mounting medium containing DAPI (VECTOR Laboratories, Newark, CA) and analyzed. Images were acquired on a Olympus FluoView FV1000 BX2 Upright Confocal microscope. To assess vessel leakage, immunostaining of retinal cryosections was performed on eyes isolated from 4% paraformaldehyde (PFA) perfused animals using Isolectin B4 stain and antibody against mouse Albumin (ab207327, Abcam).

### 2.8 RNAscope in‐situ hybridization

To identify mRNA molecules, RNAscope was carried out on formalin‐fixed paraffin embedded (FFPE) *Ercc1* ^*−/Δ*^ and WT control retinal slices. Sections of 5 μm thickness were obtained from FFPE blocks. The experiment included one positive control probe (RNAscope 3‐plex Positive control probe), one negative control probe (RNAscope 3‐plex Negative control probe), and two distinct probes targeting genes of interest: *p16* ^*Ink4a*^ (Cdkn2a‐tv2–NM_001040654.1) and *p21* ^*Cip1*^ (Cdkn1a–NM_007669.4). In situ hybridization followed the protocol outlined in the RNAscope Multiplex Fluorescent Reagent Kit v2 (Cat. no. 323100). Briefly, slides were dehydrated in 50%, 70%, and 100% ethanol for 5 min each at room temperature (RT). After a 5‐min drying step at RT, H_2_O_2_ was applied for 10 min at RT. To enhance antigen accessibility, slides were subjected to target retrieval reagent at 99°C for 15 min and Protease plus for 30 min at RT. C2 probes were diluted in C1 probes at a 1:50 ratio and then incubated on the slides for 2 h at 40°C. Detection of C1 probes was achieved using Opal 690, while C2 probes were detected with Opal Polaris 780. To minimize autofluorescence caused by lipofuscin or other protein aggregates, slides were treated with TrueBlack Plus (Biotium, 23,014) for 10 min at RT. Prior to mounting, DAPI was added to stain the nuclei. The images were acquired using Nikon AX R microscope.

### 2.9 Staining of RPE flat mount

After euthanization of mice, the eyes were collected and fixed in 4% paraformaldehyde for 30 min. The RPE/choroid complex was incubated in blocking buffer (10% serum in PBS) for 1 h at room temperature followed by a 1 h incubation with Alexa Fluor 594 Phalloidin in antibody buffer (2% serum in phosphate buffered saline (PBS)) (1:100, Thermo‐Fisher Scientific) to enable visualization of cell shape and size in order to analyze morphological changes of the RPE. After washing with PBS (0.1 M, pH 7.4), the RPE/choroid complex was incubated with Hoechst33342 (Thermo‐Fisher scientific) for 15 min. After washing with PBS, the tissue was mounted with ProLong Gold Antifade Mountant (Invitrogen) and imaged by confocal microscopy (Olympus FluoView FV1000 BX2 Upright Confocal). Z‐stack confocal images of central to mid‐peripheral area were acquired and analyzed using FIJI software. The retinal flat mounts were prepared from six mice per genotype.

### 2.10 Staining of retinal wholemount

For retinal vasculature staining, the mouse eyes were enucleated and immersed in formalin overnight at 4°C. Afterwards, the globes were rinsed with PBS. The anterior chamber, lens, and vitreous were trimmed off. The retinas were carefully separated from the eyecup and the posterior eye segment containing the sclera–choroid complex. The retinas were dissected into quarters by four radial cuts and were incubated in AlexaFluor 488 Isolectin GS B4 (Thermo‐Fisher scientific) for 1 h at room temperature. After washing, the tissues were flat mounted with ProLong Gold Antifade Mountant (Invitrogen) and imaged using confocal microscopy (Olympus FluoView FV1000 BX2 Upright Confocal). Z‐stack confocal images were taken. Three mice per genotype were used for this experiment.

### 2.11 Measurement of oxygen consumption and glycolysis in RPE

RPE cells from mouse eyes were isolated and cultured to analyze the mitochondrial function and glycolytic rate. For RPE isolation, the eyecups were digested with 2% dispase for 45 min at 37°C, and the RPE gently brushed off from the choroid (Go et al., 2020; Shang et al., 2018). Cells were washed, seeded in 96‐well XF microplates (Agilent technologies), and cultured in DMEM: F12 (Thermo‐Fisher scientific) supplemented with 10% fetal bovine serum, 1% penicillin/streptomycin, and 1× minimal essential medium with nonessential amino acids. Cells were allowed to grow for 5–7 days at atmospheric oxygen tension (20% Oxygen). Thereafter, the Mitostress test and the glycolytic rates (Agilent technologies) were measured according to manufacturer's instructions. The data were analyzed using Wave software (Agilent Technologies). A total of three independent replicates per genotype were used for this experiment.

### 2.12 Mitochondria and mitophagy staining of RPE cells

RPE cells were isolated from mouse eyes as described above and seeded in 96 well plates (Corning, flat clear bottom, black plate). Cells were grown at 37°C with 5% CO_2_ for 5–7 days and stained with Mitotracker Green FM (Invitrogen) or Mtphagy dye (Dojindo Laboratories) according to manufacturer's instructions. The cells were incubated with 300 nM Mitotracker Green in HBSS along with Hoechst 33342 for 15 min at 37°C or Mtphagy dye at 1:1000 dilution in HBSS along with Hoechst 33342 for 30 min at 37°C. After the incubation of the cells Mtphagy dye, mitophagy was induced by treatment of 1 μM CCCP for 8 h. Then the cells were washed with PBS, and the fluorescent intensity measured using a Varioskan LUX Multimode Microplate Reader (Thermo‐Fisher Scientific). The excitation/emission wavelengths used were 490/516 and 530/700 nm for Mitotracker Green and Mtphagy dye, respectively. Images were acquired using a Nikon A1Rsi HD confocal microscope. A total of three independent replicates per genotype were examined.

### 2.13 C_12_FDG staining

Primary RPE were isolated as described above and seeded in 96 well plates (Corning, flat clear bottom, black plate). The cells were stained with C_12_FDG (Setareh Biotech), a lipophilic green, fluorescent substrate, to detect lysosomal β‐galactosidase activity. Cells were grown at 37°C with 5% CO_2_ for 5–7 days. The cells were treated with 100 nM Bafilomycin‐A1 (Cell Signaling Technology) in growth medium for 1 h at 37°C. 10 μM of C_12_FDG was added and incubated for 2 h at 37°C. Cells were washed with PBS and incubated with Hoechst 33342 for 15 min at 37°C. Then the cells were washed with PBS and images were acquired using a Cytation 5 (Agilent Technologies). A total of three independent biological replicates were analyzed.

### 2.14 Western blot

PINK1, PARKIN, and TOMM20 expression were measured in the RPE layer of *Ercc1* ^*−/Δ*^ and WT mice via immunoblotting. RPE/Choroid layers were lysed on ice for 30 min in RIPA lysis buffer supplemented with protease inhibitors (Thermo Fisher Scientific). Samples were centrifuged (30 min at 12000 g at 4°C), and the protein concentration in the supernatant was determined using a BCA assay (Thermo Fischer Scientific). Fifteen microgram of protein were loaded onto a 4%–20% Criterion TGX Precast Midi Protein Gel (Bio‐Rad Laboratories) and electrophoresed. Proteins were transferred to a PVDF membrane, blocked, and probed with antibodies against PINK1 (1:1000, rabbit monoclonal, Cell Signaling Technologies), PARKIN (1:1000, rabbit monoclonal, Proteintech), TOMM20 (1:1000, mouse monoclonal, Abcam), and α‐tubulin (1:2000, mouse monoclonal, Abcam) overnight at 4°C. After washing, secondary peroxidase‐labeled antibodies were applied, followed by chemiluminescence detection (Thermo Fisher Scientific). Images were captured using iBright (Thermo Fisher Scientific), and GelAnalyzer software was used for quantification. Relative signal intensity was normalized to α‐tubulin, with three independent replicates per genotype analyzed.

## 3 RESULTS

### 3.1 *Ercc1* ^*−/Δ*^ mice have impaired vision

**FIGURE 1:**
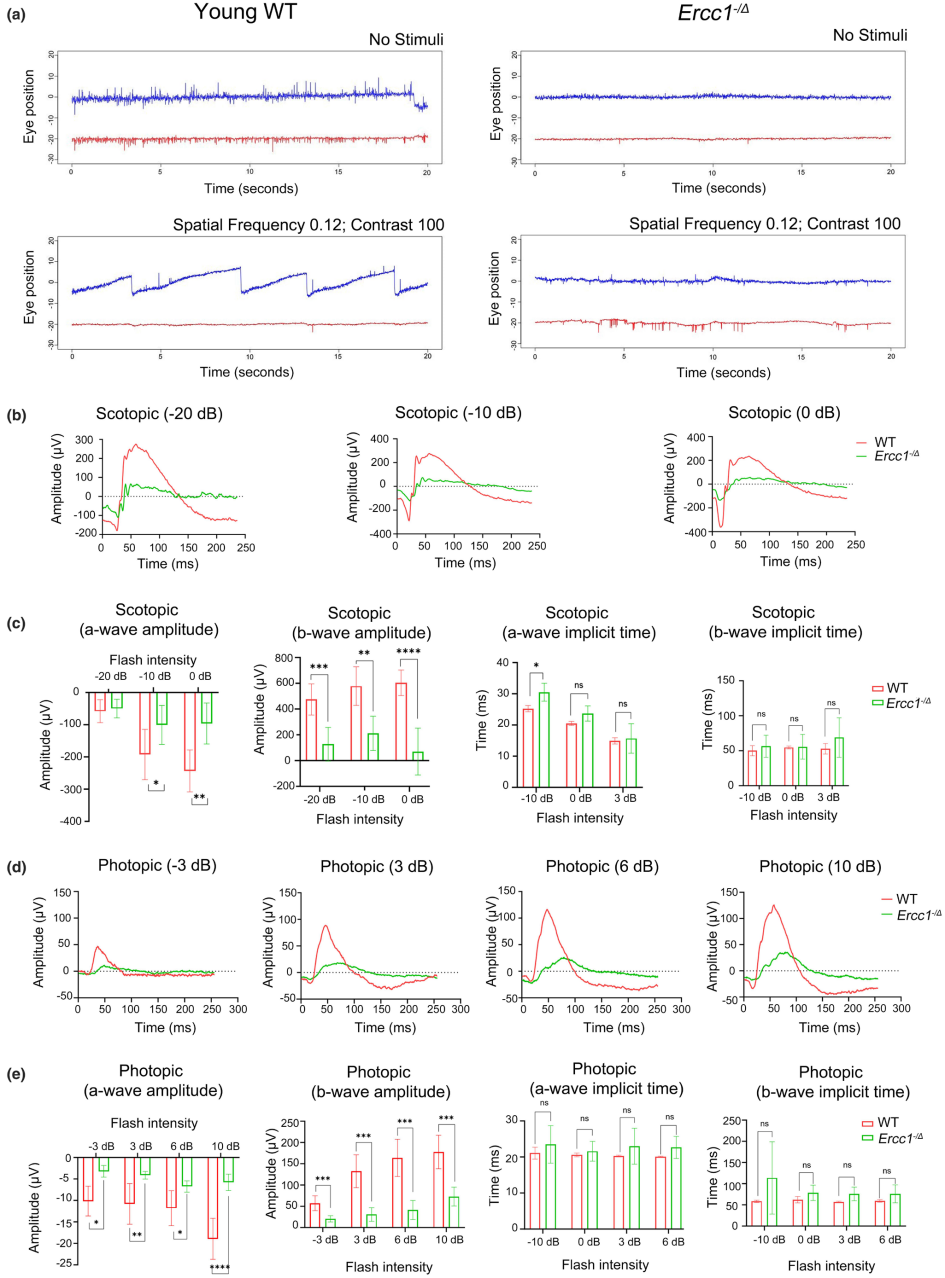
*Ercc1* ^*−/Δ*^ mice show impaired vision. (a) Representative images for optokinetic response (OKR) of an *Ercc1* ^*−/Δ*^ mouse and WT control at 4‐months‐of‐age. The graphs show eye position in horizontal (blue line) and vertical (red line) components as a function of time for 20 s. Eye movements were analyzed in the absence of stimulus (top) or with a stimulus with a spatial frequency of 0.12 and a contrast of 100 (bottom). (b) Representative scotopic electroretinogram (ERG) using a series of light intensities (−20 dB, −10 dB, 0 dB) on an *Ercc1* ^*−/Δ*^ mouse and WT control at 3‐months‐of‐age. (c) A‐wave and b‐wave amplitude and implicit time of scotopic ERG (*n* = 3 mice per genotype). (d) Representative photopic ERG using a series of light intensities (−3 dB, 3 dB, 6 dB, 10 dB) in *Ercc1* ^*−/Δ*^ and age‐matched WT mice. (e) A‐wave and b‐wave amplitude and implicit time of photopic ERG (*n* = 3 mice per genotype). In figure c and e, the results are represented as mean ± SD and statistical significance was determined by an unpaired two‐tailed Student's *t*‐test, *p < 0.03, **p < 0.002, ***p < 0.0002, ****p < 0.0001, ns, not significant.

The vision of *Ercc1* ^*−/Δ*^ and age‐matched WT littermates was measured using optokinetic response (OKR) and electroretinogram (ERG). The OKR, which measures eye movements in response to visual stimuli reflecting a reflex needed to stabilize images on the retina to compensate for motion. An OKR signal is dependent upon vision. The assay revealed abnormal patterns in 4‐month‐old *Ercc1* ^*−/Δ*^ mice compared to WT controls (Figure 1a), suggesting that the mutant mice have abnormal eye movements in response to visual stimuli and potentially visual impairment. In scotopic ERGs, which measures electrical activity in the retina in response to light, mice were presented with low intensity light flashes (−20 dB, −10 dB, 0 dB) to induce rod activation and examine the function of rod photoreceptor and downstream retinal cells. The amplitude with low intensity flashes were significantly reduced in 3‐month‐old *Ercc1* ^*−/Δ*^ mice relative to age‐matched WT controls (Figure 1b). The a‐wave amplitude is a measure of initial response of photoreceptors to a brief flash of light, and the b‐wave is a measure of the response of downstream retinal neurons, including bipolar cells, to photoreceptor stimulation. Both the a‐wave and b‐wave amplitudes in the scotopic ERG were significantly reduced at all three light intensities (Figure 1c). This indicates that the rod function as well as other retinal neuron function were greatly reduced in the *Ercc1* ^*−/Δ*^ mice relative to age‐matched WT controls. To evaluate cone function, the photopic ERG was performed with high intensity light flashes (−3 dB, 3 dB, 6 dB, 10 dB) after a period of light stimulation. Similar to the scotopic ERG, the a‐wave and b‐wave amplitudes were significantly reduced in the photopic ERG (Figure 1d,e). The a‐wave implicit time (time‐to‐peak) can be measured by calculating elapsed time between *t* = 0 and the time point at the lowest point on the trace. The b‐wave implicit time can be measured by calculating elapsed time between the bottom of the a‐wave and the top of the b‐wave. No significant changes were observed in a‐wave and b‐wave implicit time of scotopic and photopic ERG (Figure 1c,e). These results demonstrate that the function of both photoreceptors (rods and cones) as well as other retinal neurons were severely impaired in *Ercc1* ^*−/Δ*^ mice at 3–4 months‐of‐age.

### 3.2 Deficiency of *Ercc1* affects retinal vasculature

**FIGURE 2:**
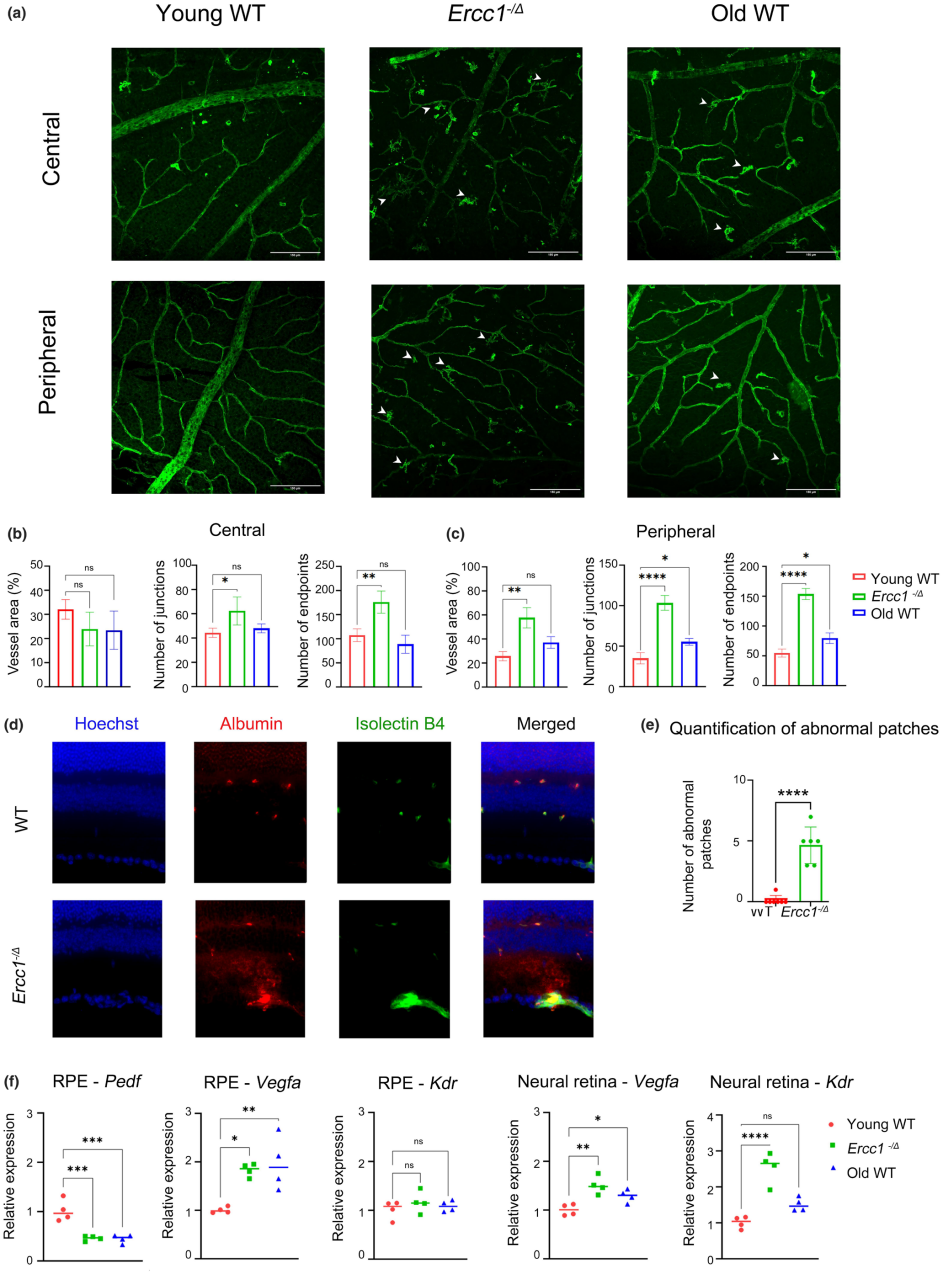
Deficiency of *Ercc1* expression affects retinal vasculature. (a) Representative images of retinal vasculature visualized by Isolectin B4 staining of retinal whole mounts from an *Ercc1* ^−/Δ^ mouse and WT control at 4‐months‐of‐age, as well as an aged WT mouse (30‐month‐old). Images were taken in the superficial vascular plexus of the central and peripheral retina. Arrows indicate pathological neovascularization in *Ercc1* ^*−/Δ*^ and aged WT mouse retina. Scale bar = 150 μm. (b) Graphical representations of the vascular quantification conducted within the central area of the superficial plexus using AngioTool. Vessel area percentage, number of junctions, number of endpoints were measured in *n* = 4 mice. The results are represented as mean ± SD and statistical significance was determined by one‐way ANOVA, *p < 0.03, **p < 0.002, ns, not significant. (c) Graphical representation of the vascular quantification in the peripheral area of the superficial plexus using AngioTool. Vessel area percentage, number of junctions, number of endpoints were measured in *n* = 4 mice. The results are represented as mean ± SD and statistical significance was determined by one‐way ANOVA, *p < 0.03, **p < 0.002, ****p < 0.0001, ns, not significant. (d) Retinal cryosections of eyes isolated from 4% PFA perfused animals were stained with stain against Isolectin B4 and antibody against mouse Albumin. Representative images showing sections from WT and *Ercc1* ^−/Δ^ animals where only mutant animals show Isolectin/Albumin patches with visible Albumin spill in the retinal tissue. (e) Quantification of Isolectin/Albumin patches per section in WT and *Ercc1* ^−/Δ^ animals. (f) Expression of genes related to angiogenesis (*Pedf*, *Vegfa*, *Kdr*) in the RPE/choroid and the neural retina from *Ercc1* ^*−/Δ*^ mice and WT controls at 4‐months‐of‐age, as well as aged WT mice at 30‐months of age (*n* = 4 per genotype). *Gapdh* was used as housekeeping gene control. The results are represented as mean ± SD and statistical significance was determined by one‐way ANOVA, *p < 0.03, **p < 0.002, ***p < 0.0002, ****p < 0.0001, ns, not significant.

Vascular abnormalities of the retina is a characteristic feature of aging and AMD. To determine how DNA damage affects the vasculature, retinal flat mounts were stained with Isolectin B4 (Figure 2a). This revealed abnormal vascularization in 4‐month‐old *Ercc1* ^*−/Δ*^ retina and 30‐month‐old WT mice relative to 4‐month‐old WT mice. Vessel area, number of branches and endpoints were increased in *Ercc1* ^*−/Δ*^ animals compared to age‐matched WT animals (Figure 2b,c). To assess the potential vessel leakage, immunostaining of retinal cryosections was performed using Isolectin B4 stain and antibody against mouse Albumin (Figure 2d). Only sections from mutant animals displayed aberrant Isolectin/Albumin patches with visible Albumin signal spilled in the retina, showing the points of loss of vessel integrity (Figure 2e). The expression of genes related to angiogenesis were measured in RPE/choroid as well as neural retina by qRT‐PCR (Figure 2f). The RPE/choroid isolated from *Ercc1* ^*−/Δ*^ mice had significantly reduced levels of anti‐angiogenic gene *Pedf/Serpinf1* relative to age‐matched WT mice, along with increased expression of the pro‐angiogenic factor *Vegfa*, similar to 30‐month‐old WT mice. Although there was an elevated expression of *Vegfa*, no significant change in the expression of the VEGF receptor 2 (*Kdr*) was detected in RPE/choroid. *Vegfa* and *Kdr* gene expression were upregulated in the neural retinal of the mutant mice, and to a lesser extent in the old WT mice, relative to young adult WT mice. These results indicate that genotoxic stress and senescence in the RPE and neural retina promote pathological angiogenesis, an age‐related feature impacting vision and retinal degeneration.

### 3.3 *Ercc1* deficiency alters retinal structure and increases senescence markers in neural retina

**FIGURE 3:**
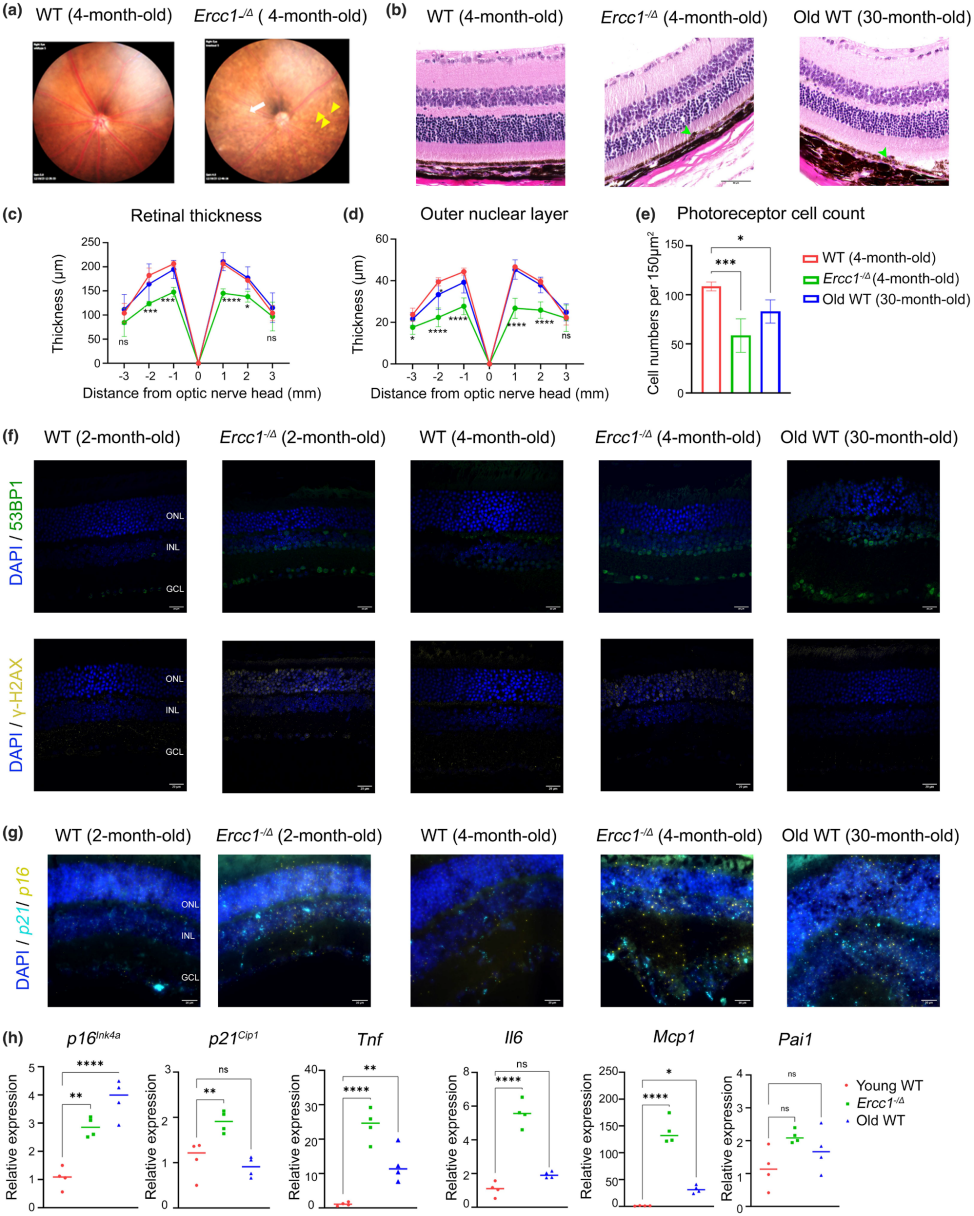
*Ercc1* deficiency alters retinal structure and increases senescence markers in neural retina. (a) Representative images of color fundus photography of *Ercc1* ^*−/Δ*^ mouse and WT control at 3‐months‐of‐age to assess retinal integrity. Acquired images show visible changes in retina thickness and lower level of blood‐dependent coloration in the mutant retina. The RPE shows signs of accelerated aging such as degeneration (white arrow) and accumulation of deposits (yellow arrowheads). (b) Representative images of H&E‐stained sections of retinas from an *Ercc1* ^*−/Δ*^ mouse and WT control at 4‐months‐of‐age, as well as an aged WT mouse at 30‐months‐of‐age demonstrating degeneration of the retina in *Ercc1* ^*−/Δ*^ mice. Arrows indicate subretinal drusenoid deposits in *Ercc1* ^*−/Δ*^ and aged WT mouse. Images were taken close to the optic nerve head. Scale bar = 50 μm. (c) Retinal thickness was measured at 6 consecutive points (1000, 2000, 3000 microns to the left and right of the optic nerve head) and plotted as a spider plot. Retinal sections (*n* = 3 retinal sections per eye) from *n* = 4 mice for each genotype were measured. The results are represented as mean ± SD and statistical significance was determined by two‐way ANOVA, *p < 0.03, ****p < 0.0001, ns, not significant. (d) Outer nuclear layer thickness was measured at 6 consecutive points (1000, 2000, 3000 microns to the left and right of the optic nerve head) and plotted as a spider plot. Retinal sections (*n* = 3 retinal sections per eye) from *n* = 4 mice for each genotype were measured. The results are represented as mean ± SD and statistical significance was determined by two‐way ANOVA, *p < 0.03, **p < 0.002, ***p < 0.0002, ****p < 0.0001, ns, not significant. (e) Photoreceptor cell numbers were counted per 150 μm^2^ area in the retina. The cells were counted close to the optic nerve head from *n* = 4 mice for each genotype. Each bar represents mean ± SD statistical significance was determined by one‐way ANOVA, *p < 0.03, ***p < 0.0002. (f) Representative images illustrating the immunostaining of DNA damage markers 53BP1 (green) and γ‐H2Ax (yellow) in retinal sections of *Ercc1* ^*−/Δ*^ mice and WT controls at 2‐months and 4‐months, as well as aged WT mice at 30‐months‐of‐age (*n* = 3 per group). The retinal layers are labelled as follows: GCL, Ganglion cell layer; INL, Inner nuclear layer; ONL, Outer nuclear layer. Scale bar = 20 μm. (g) Representative images illustrating the RNAscope detection of senescence markers–*p16* (yellow) *p21* (cyan) mRNA in retinal sections of *Ercc1* ^*−/Δ*^ mice and WT controls at 2‐months, 4‐months, as well as aged WT mice at 30‐months‐of‐age (*n* = 3 per group). The retinal layers are labelled as follows: GCL, Ganglion cell layer; INL, Inner nuclear layer; ONL, Outer nuclear layer. Scale bar = 20 μm. (h) The expression of senescence biomarkers (*p16* ^*Ink4a*^ and *p21* ^*Cip1*^) and SASP factors (*Tnf*, *Il6*, *Mcp1*, *Pai1*) were measured by qRT‐PCR in neural retina of *Ercc1* ^−*/Δ*^ mice and WT controls at 4‐months‐of‐age, as well as aged WT mice at 30‐months‐of‐age (*n* = 4 per group). *Gapdh* was used as internal control. The results are represented as mean ± SD and statistical significance was determined by one‐way ANOVA, *p < 0.03, **p < 0.002, ***p < 0.0002, ****p < 0.0001, ns, not significant.

Fundus photography was performed to visualize retinal deposits and degeneration. *Ercc1* ^*−/Δ*^ mice displayed signs of accelerated aging, such as RPE degeneration and the accumulation of deposits in the subretinal region by 4‐months of age (Figure 3a). Next, we examined the neural retina histology in 4‐month‐old *Ercc1* ^*−/Δ*^ mice. H&E staining of retinal cross sections from *Ercc1* ^*−/Δ*^ and 30‐month‐old WT mice revealed subretinal deposits adjacent to the RPE (Figure 3b), a characteristic of aging and AMD. The columnar organization of the outer nuclear layer photoreceptor cell nuclei was diminished. The retinal thickness (Figure 3c), the thickness of the outer nuclear layer (Figure 3d), as well as photoreceptor cell nuclei counts (Figure 3e) were significantly reduced in the retina of *Ercc1* ^*−/Δ*^ mice and aged WT (30‐months‐old) mice relative to 4‐month‐old WT mice.

Retinal sections were stained with antibodies to DNA damage markers 53BP1 and γ‐H2Ax (Figure 3f). Remarkably, 53BP1 staining was elevated even in 2‐month‐old *Ercc1* ^*−/Δ*^ mice, particularly prominent in the inner nuclear layer and ganglion cell layer. In contrast, γ‐H2Asx staining showed increased intensity in the ONL. These results parallel observations in aged WT mice (30‐months‐old), suggesting that spontaneous, endogenous DNA damage contributes retinal aging. To explore whether DNA damage triggers senescence in the neural retina, we utilized the in‐situ hybridization RNAscope to assess the spatial expression of senescence markers, namely *p16* ^*Ink4a*^ and *p21* ^*Cip1*^. Interestingly, both senescence markers were elevated across all retinal layers in both 2 and 4‐month‐old *Ercc1* ^*−/Δ*^ mice, resembling the expression patterns observed in aged WT mice (30‐months‐old) (Figure 3g).

The senescence markers (*p16* ^*Ink4a*^, *p21* ^*Cip1*^) and SASP factors (*Tnf*, *Il6*, *Mcp1*, *Pai1*) were also measured using qRT‐PCR (Figure 3h). The neural retina of 4‐month‐old *Ercc1* ^*−/Δ*^ mice have significantly elevated expression of *p16* ^*Ink4a*^, *p21* ^*Cip1*^, *Tnf*, *Il6*, and *Mcp1* compared to age‐matched WT mice. The expression of p*16* ^*Ink4a*^, *Tnf*, and *Mcp1* was significantly elevated in the neural retina of 30‐month‐old WT mice compared to 4‐month‐old WT animals, but not *p21* ^*Cip1*^ or *Il6* (Figure 3h). Together, these data suggest that senescence occurs in the retina as a consequence of aging and that spontaneous DNA damage can drive expression of markers of senescence in the neural retina.

### 3.4 Genetic depletion of *Ercc1* results in cellular senescence of RPE

**FIGURE 4:**
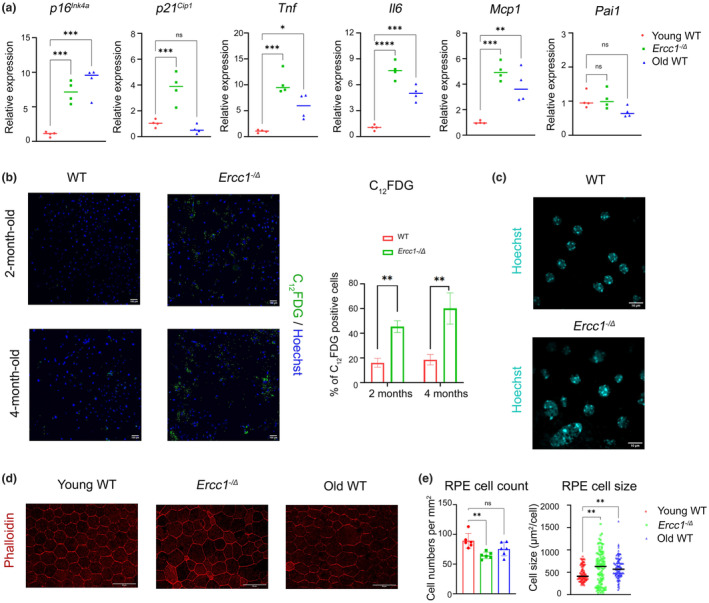
Genetic depletion of *Ercc1* results in cellular senescence of RPE. (a) Expression of senescence biomarkers (*p16* ^*Ink4a*^ and *p21* ^*Cip1*^) and SASP factors (*Tnf*, *Il6*, *Mcp1*, *Pai1*) were measured by qRT‐PCR in RPE isolated from *Ercc1* ^*−/Δ*^ mice and WT controls at 4‐months‐of‐age, as well as old WT mice at 30‐months‐of‐age (*n* = 4 per group). *Gapdh* was used as a housekeeping gene internal control. The results are represented as mean ± SD and statistical significance was determined by one‐way ANOVA, *p < 0.03, **p < 0.002, ***p < 0.0002, ****p < 0.0001, ns, not significant. (b) Representative images of C_12_FDG staining to detect SA‐β‐gal activity in primary cultured RPE cells from an *Ercc1* ^−*/Δ*^ mouse and WT control at 2 and 4‐months‐of‐age. Scale bar = 100 μm. The bar graph represents the percentage of C_12_FDG positive cells in both 2‐ and 4‐month age groups. The results are represented as mean ± SD and statistical significance was determined by an unpaired two‐tailed Student's *t*‐test, **p < 0.002. (c) Representative images of nuclei staining (Hoechst) in RPE flat mounts from an *Ercc1* ^−/Δ^ mouse and WT control at 4‐months‐of‐age, illustrating enlarged nuclei in the mutant animal. Scale bar = 10 μm. (d) Representative images of F‐actin staining (phalloidin) to detect morphology of RPE cells in flat mounts of an *Ercc1* ^−/Δ^ mouse and WT control at 4‐months‐of‐age as well as an old WT mouse at 30‐months‐of‐age. Scale bar = 50 μm. (e) RPE cell numbers per mm^2^ and cell size (μm^2^/ cell) were measured in the central to mid‐peripheral area of RPE flat mounts using FIJI software (*n* = 6 biological replicates). The results are represented as mean ± SD and statistical significance was determined by one‐way ANOVA, **p < 0.002, ns, not significant.

To determine whether spontaneous endogenous DNA damage drives cellular senescence of RPE, the expression of senescence markers (*p16* ^*Ink4a*^, *p21* ^*Cip1*^) and SASP factors (*Tnf*, *Il6*, *Mcp1*, and *Pai1*) were measured by qRT‐PCR (Figure 4a) in RPE isolated from 4‐month‐old *Ercc1* ^*−/Δ*^ mice and compared to age‐matched WT mice. RPE from old WT (30‐month‐old) mice were also examined to determine if RPE senescence occurs with normal aging. The levels of all of the senescence markers were significantly elevated in *Ercc1* ^*−/Δ*^ mouse RPE, compared to age‐matched controls, with the exception of *Pai1* (Figure 4a). In old WT mice, the senescence endpoints were also significantly elevated except *p21* ^*Cip1*^ and *Pai1*. Cellular senescence was confirmed by C_12_FDG staining of primary RPE cells isolated from 2‐month and 4‐month‐old WT and *Ercc1* ^*−/Δ*^ mice to measure senescence‐associated β‐galactosidase activity (Figure 4b). Staining was greater in *Ercc1* ^*−/Δ*^ RPE than that seen in age‐matched WT RPE. In RPE flat mounts, the nuclear size was enlarged and more heterogeneous in the 4‐month‐old DNA repair deficient mice (Figure 4c). F‐actin staining of RPE flat mounts revealed heterogenous cell sizes and irregular cell morphology in *Ercc1* ^*−/Δ*^ RPE relative to age‐matched 4‐month‐old WT mouse (Figure 4d). The RPE from 30‐month‐old WT mice also contained enlarged cells. The cell count per area and cell sizes were significantly altered in the *Ercc1* ^*−/Δ*^ and old WT mice relative to young WT controls (Figure 4e). These results demonstrate that RPE, like neural retina, is vulnerable to endogenous DNA damage, and this genotoxic stress promotes cellular senescence and cell loss in the RPE.

### 3.5 Genetic mutation of *Ercc1* leads to glycolytic switch in RPE

**FIGURE 5:**
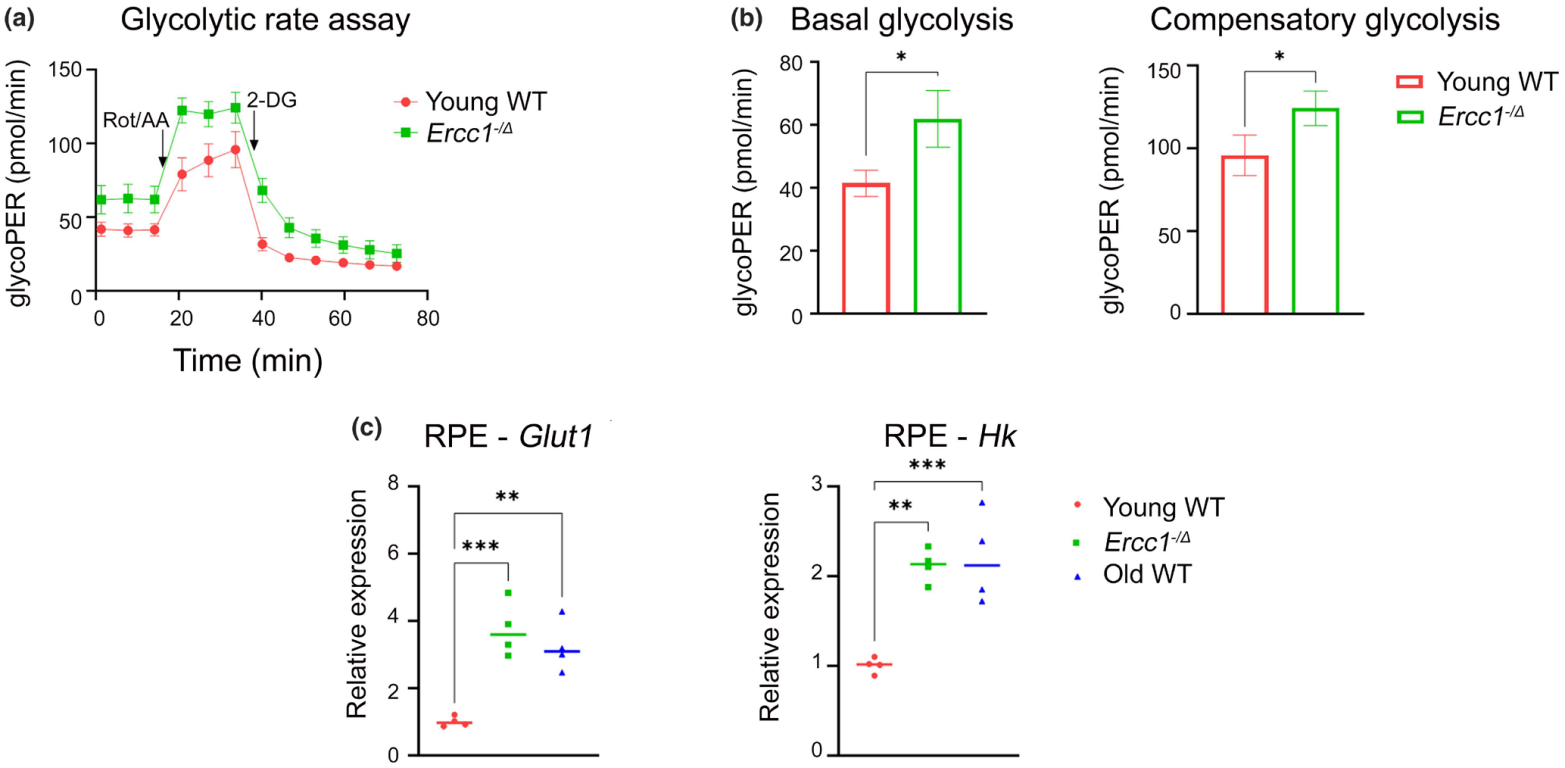
Genetic mutation of *Ercc1* leads to glycolytic switch of RPE. (a) Measurement of glycolysis capacity by Seahorse assay using primary mouse RPE cells established from *Ercc1* ^*−/Δ*^ mice and WT controls at 4‐months‐of‐age (*n* = 3 biological replicates). (b) Basal glycolysis and compensatory glycolysis calculated from glycolytic rate assay results using Wave software in cultured primary RPE cells from *Ercc1* ^*−/Δ*^ mice and WT controls at 4‐months‐of‐age (*n* = 3 biological replicates). The results are represented as mean ± SD and statistical significance was determined by an unpaired two‐tailed Student's *t*‐test, *p < 0.03. (c) The expression of glycolytic genes (*Glut1* and *Hk*) measured using qRT‐PCR in RPE from *Ercc1* ^*−/Δ*^ mice and WT controls at 4‐months‐of‐age, as well as old WT mice at 30‐months‐of‐age (*n* = 4 per group). *Gapdh* was used as housekeeping gene control. The results are represented as mean ± SD and statistical significance was determined by one‐way ANOVA, **p < 0.002, ***p < 0.0002.

In AMD, the mitochondrial dysfunction in RPE triggers the cell to use glucose from the choroid and reliance on glycolysis for energy (Fisher & Ferrington, 2018). This reduces the level of glucose transported to the photoreceptors. This metabolic shift can leads to death of both photoreceptors and RPE (Fisher & Ferrington, 2018). As photoreceptor loss (Figure 3d) and retinal dysfunction (Figure 1) were observed in *Ercc1* ^*−/Δ*^ mice, the glycolytic rate was measured in primary RPE isolated from 4‐month‐old *Ercc1* ^*−/Δ*^ mice and age‐matched WT controls (Figure 5a,b). Basal glycolysis and compensatory glycolysis levels were significantly increased in RPE cells from *Ercc1* ^*−/Δ*^ mice relative to controls. This shows that RPE cells from mutant mice rely on glycolysis as an energy source to a greater extent than age‐matched WT mice. In addition, we measured gene expression of glucose transporter‐1 (*Glut1*) and hexokinase (*Hk*) in 4‐month‐old WT, *Ercc1* ^*−/Δ*^, and 30‐month‐old WT mouse RPE (Figure 5c). These genes were upregulated in the *Ercc1* ^*−/Δ*^ and old WT mice compared to young adult WT controls. These results suggest that endogenous DNA damage in the RPE contributes to metabolic changes characteristic of senescent cells and aging, contributing to age‐related loss of vision.

### 3.6 Genetic mutation of *Ercc1* leads to mitochondrial dysfunction in RPE

**FIGURE 6:**
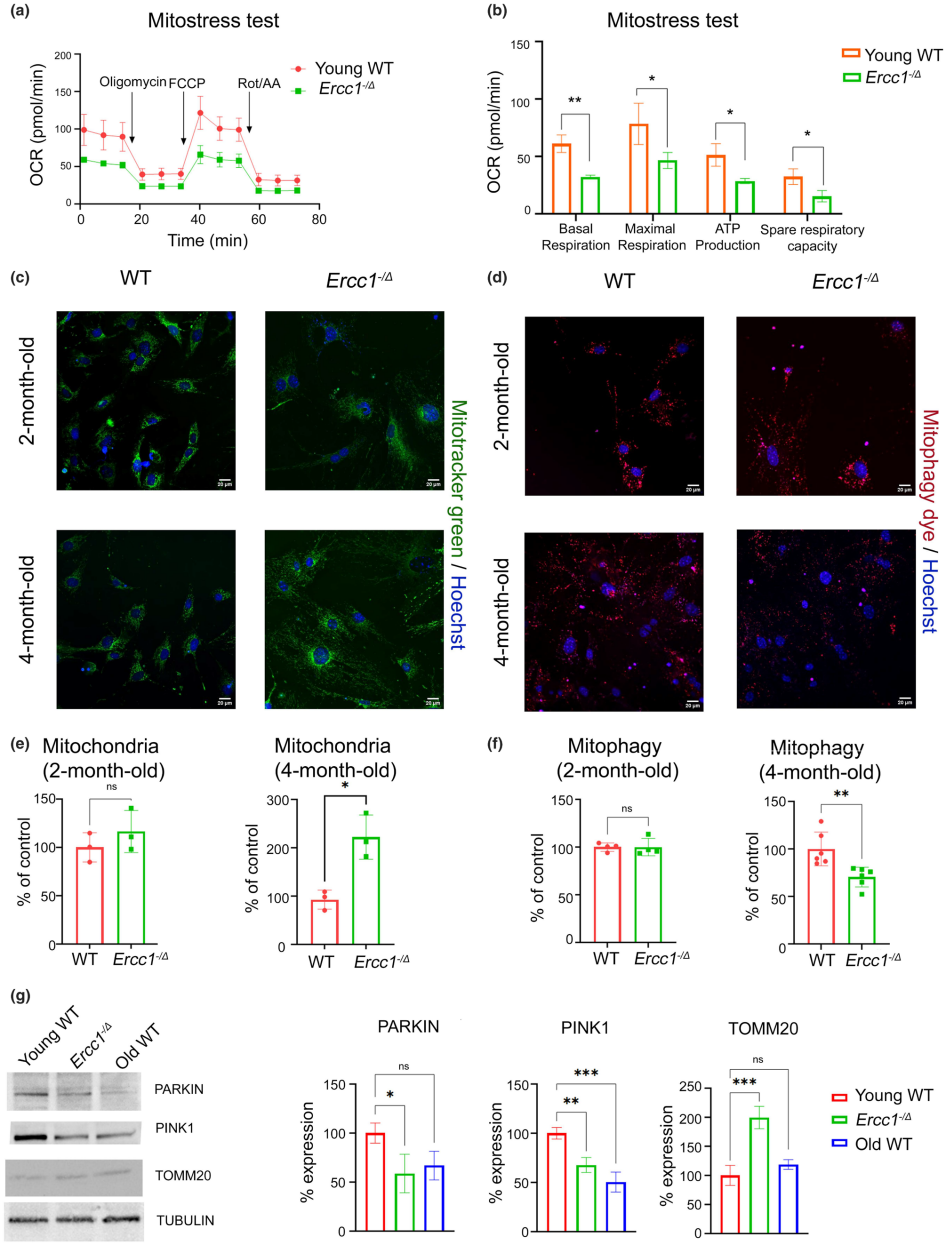
Genetic mutation of *Ercc1* leads to mitochondrial dysfunction in RPE. (a) Mitochondrial function was measured by Mito stress test in primary mouse RPE cultures established from *Ercc1* ^−*/Δ*^ mice and WT controls at 4‐months‐of‐age (*n* = 3 mice per genotype) using a Seahorse analyzer. (b) Basal respiration, maximum respiration, ATP production, spare respiratory capacity was calculated from the Mito stress test in panel A using Wave software. The results are represented as mean ± SD and statistical significance was determined by an unpaired two‐tailed Student's *t*‐test, *p < 0.03, **p < 0.002. (c) Representative images of mitochondrial staining using MitoTracker green in cultured primary RPE cells from an *Ercc1* ^−*/Δ*^ mouse and a WT control at 2 and 4‐months‐of‐age. Scale bar = 20 μm. (d) Mitochondria content measured by fluorescence detection of a mitochondria‐specific dye in primary RPE from *Ercc1* ^*−/Δ*^ mice and age‐matched WT littermates (data from panel C; *n* = 3 biological replicates). The results are represented as mean ± SD and statistical significance was determined by an unpaired two‐tailed Student's *t*‐test, *p < 0.03, ns, not significant. (e) Representative images of mitophagy staining using MtPhagy dye in cultured primary RPE cells from an *Ercc1* ^−*/Δ*^ mouse and a WT control at 2 and 4‐months‐of‐age. Scale bar = 20 μm. (f) Mitophagy was measured by fluorescence detection of mitophagy specific dye in primary RPE from *Ercc1* ^*−/Δ*^ mice and age‐matched WT littermates (data from panel D; *n* = 3 biological replicates). The results are represented as mean ± SD and statistical significance was determined by an unpaired two‐tailed Student's *t*‐test, **p < 0.002; ns, not significant. (g) Expression levels of proteins involved in mitophagy (PINK1 and PARKIN), and protein related to mitochondrial mass (TOMM20) were measured by western blot in RPE from *Ercc1* ^*−/Δ*^ mice WT animals at 4‐months‐of‐age, as well as old WT mice at 30‐months‐of‐age (*n* = 3 per group). Tubulin was used as a housekeeping control. The results are represented as mean ± SD and statistical significance was determined by a one‐way ANOVA, *p < 0.03, ***p < 0.0002, ns, not significant.

Reduced mitochondrial ATP production and altered mitochondrial dynamics are well known characteristics of aging RPE (Tong et al., 2022). To assess mitochondrial function of RPE, oxygen consumption rate (OCR) was measured in primary RPE cultures under conditions of metabolic challenge (Figure 6a,b). RPE from 4‐month‐old *Ercc1* ^*−/Δ*^ mice had decreased OCR compared to age‐matched WT controls. The basal respiration, maximal respiration, ATP production, and spare respiratory capacity were significantly decreased in *Ercc1* ^*−/Δ*^ RPE relative to controls. To check whether decreased mitochondrial function had any association with mitochondrial mass, the primary RPE cells were stained with Mitotracker green (Figure 6c). Interestingly, mitochondrial mass was found to be increased in 4‐month‐old *Ercc1* ^*−/Δ*^ RPE cells compared to WT controls (Figure 6c,d), whereas no such elevation was observed in 2‐month‐old *Ercc1* ^*−/Δ*^ RPE cells. This observation was further validated by western blot analysis of the mitochondrial protein TOMM20, which was elevated levels in 4‐month‐old *Ercc1* ^*−/Δ*^ but not in aged WT (30‐months‐old) RPE. Mitophagy, a crucial quality control process responsible for eliminating dysfunctional mitochondria, was then investigated to determine if dysregulation contributed to the heightened mitochondrial mass. RPE primary cells were stained with Mtphagy dye for this purpose (Figure 6e,f). While mitophagy levels remained unaltered in 2‐month‐old *Ercc1* ^*−/Δ*^ primary RPE cells, a significant reduction was observed in 4‐month‐old *Ercc1* ^*−/Δ*^ primary RPE cells. This reduction was further supported by western blot analysis of mitophagy proteins such as PINK1 and PARKIN, which were notably downregulated in the RPE of 4‐month‐old *Ercc1* ^*−/Δ*^ (Figure 6g).

Overall, these results demonstrate that endogenous nuclear DNA damage, if not repaired, can drive accumulation of dysfunctional mitochondria, likely due to impaired mitophagy, similar to what occurs in the RPE with aging. This is consistent with previous studies reporting mitochondrial and mitophagic dysfunction downstream of nuclear genotoxic stress in other organs (Fang et al., 2014; Robinson et al., 2018).

## 4 DISCUSSION

In this study, we sought to determine if spontaneous endogenous DNA damage is a driver of retinal degeneration and loss of vision, offering novel preclinical models and potentially new avenues for interventions to treat the devastating age‐related disease AMD. It was previously reported that DNA repair deficient *Ercc1* ^*−/Δ*^ mice have impaired vision and loss of photoreceptors in the outer nuclear layer of the retina (Spoor et al., 2012). Consistent with that observation, we found *Ercc1* ^*−/Δ*^ mice have abnormal ERG and OKR by the age of 3–4 months‐of‐age. ERG (Tong et al., 2022) and OKR abnormalities (Sugita et al., 2020) are common features reported in aged mice. Furthermore, mice missing the base excision repair (BER) enzyme uracil‐DNA‐glycosylase, which excises deaminated cytosines, have an abnormal ERG response (Lipski et al., 2007). Similarly, decreased expression of the DNA glycosylase *hOGG1*, which removes the oxidative DNA lesion 8‐oxodG, or decreased activity of hOGG1 protein in the macula are associated with the severity of AMD (Blasiak et al., 2013). The *Csb* gene is a key component of the transcription‐coupled repair (TC) subpathway of NER. Mutations in the *Csb* gene disrupt TC‐NER, leading to increased DNA damage, photoreceptor apoptosis, and premature aging of the retina (Gorgels et al., 2007). The downregulation of genes involved in the repair of DNA double‐strand breaks (*RAD51*) and polymorphisms of the BER genes *MUTYH* and *hOGG1* are observed in AMD patients (Strunnikova et al., 2005; Synowiec et al., 2012). These studies coupled with ours, implicate all major DNA repair pathways in protecting the retina from age‐related changes and AMD. This suggests that it is the downstream cellular response to genotoxic stress that drives pathophysiology in the retina.

Vision loss in AMD is associated with RPE degeneration and photoreceptor cell death. The integrity of the RPE cell monolayer is essential for maintaining normal barrier function (Chen et al., 2016; Kim et al., 2021). *Ercc1* ^*−/Δ*^ mouse RPE cells and nuclei show morphological heterogeneity in shape and size similar to age‐related changes in WT mice (Chen et al., 2016; Kim et al., 2021). Cellular hypertrophy and multinucleation may be a strategy for RPE cells to compensate for nuclear DNA damage (Chen et al., 2016). Angiogenesis in the retina and choroid is another key feature of aging retina and if left untreated contributes to blindness (Cabral et al., 2017; Dreyfuss et al., 2015). Pigment epithelium derived factor (PEDF) is a potent anti‐angiogenic factor, whereas VEGF‐A promotes neovascularization. PEDF can suppress VEGF expression levels (Wang et al., 2022). Elevated levels of VEGF‐A, both at the transcript and protein level, are observed in AMD patients with neovascularization (Frank, 1997; Kvanta et al., 1996), and anti‐VEGF is the standard of care for treating the wet form of AMD (Cabral et al., 2017; Kovach et al., 2012). *Pedf* expression is reduced, while *Vegf* is increased in the RPE/choroid of *Ercc1* ^*−/Δ*^ and old WT mice, consistent with neovascularization. The loss of blood vessel integrity was also observed in *Ercc1* ^*−/Δ*^ animals. Pro‐inflammatory cytokines, such as VEGF, and oxidative stress are known to increase vascular permeability (Campochiaro, 2013). The leakage of plasma proteins into the retina can contribute to vision problems and exacerbate inflammatory responses. Our observations also suggest that DNA damage accumulation and cellular senescence could play a role in loss of retinal vessel integrity and promoting blood vessel leakage. Senescent cells are known to secrete a variety of pro‐inflammatory cytokines, growth factors, and proteases, collectively referred to as the senescence‐associated secretory phenotype (SASP) (Campisi, 2013). The SASP can contribute to tissue dysfunction and chronic inflammation, further compromising vascular integrity. This indicates that while mice do not have a macula and therefore do not offer a perfect model of AMD, the molecular events driving AMD pathology appear to be similar, and endogenous DNA damage can trigger those events.

Evidence of DNA damage was localized in the eye of DNA repair‐deficient aged WT mice (Figure 3f). 53BP1 localized to the inner nuclear layer and ganglion cell layer of the retina in *Ercc1* ^*−/Δ*^ mice. Conversely, the levels of γ‐H2AX was elevated in the outer nuclear layer of the same mice. This distinct pattern of expression suggests differential responses to DNA damage across retinal layers. In contrast, we found abundant expression of the senescence markers *p16* ^*Ink4a*^ and *p21* ^*Cip1*^ throughout all retinal layers, even in 2‐month‐old *Ercc1* ^*−/Δ*^ mice. Our findings hint at the complexity of the DNA damage response between different cell types and its role in driving senescence and/or death.

We find elevated levels of senescence markers (*p16* ^*Ink4a*^, *p21* ^*Cip1*^, SA‐β‐gal activity) and SASP (*Tnf*, *Mcp1*, *Il6*) in RPE and neural retina of DNA repair deficient mice with partial overlap in aged WT mice (Figures 3h and 4a). The presence of senescent cells in aging eyes or with AMD has been reported (Kozlowski, 2012; Lee et al., 2021; Mishima et al., 1999; Zhu et al., 2009). The oxygen‐induced retinopathy model of AMD has increased senescent markers (*p16* ^*Ink4a*^, *p21* ^*Cip1*^ and SA‐β‐gal) and SASP, including *Vegf*, *Il1β*, *Il6*, *Tgfβ*1, and *Pai1*. In this model, senescent markers are increased in several retinal cell types, including vascular endothelia, pericytes, astrocytes, and Müller glia cells, which in turn contributes to pathologic angiogenesis (Crespo‐Garcia et al., 2021).

RPE regulates complement activation in subretinal macrophages and contributes to an immune suppressive microenvironment of the subretinal space (Luo et al., 2018). Under disease conditions or under any inflammatory insult, RPE lose their immunosuppressive properties and promote complement activation in subretinal macrophages (Luo et al., 2018). The activated macrophages secrete chemokines, proteins that regulate endothelial cell migration and matrix remodeling, all of which could directly drive neovascularization (Liu et al., 2016). These previous reports support our findings suggesting that senescent neural retinal cells and RPE can drive pathological angiogenesis in *Ercc1* ^*−/Δ*^ or aged WT mice.

The retina functions as a metabolic ecosystem, where RPE mainly depends on mitochondrial oxidative phosphorylation for energy production and transports glucose to photoreceptors, which mainly depend on aerobic glycolysis (Warburg effect) (Kanow et al., 2017). A metabolic shift of RPE from oxidative phosphorylation to glycolysis results in photoreceptor death (Kurihara et al., 2016). RPE isolated from AMD patients has impaired mitochondrial function and reduced ATP production compared to healthy subjects (Ferrington et al., 2017; Golestaneh et al., 2017; Kaarniranta et al., 2020). Damage to the nuclear genome has been shown to lead to mitochondrial dysfunction and increased ROS abundance (Robinson et al., 2018; Selfridge et al., 2001). Furthermore, cellular senescence is associated with impaired mitochondrial bioenergetics and mitochondrial dynamics (fission and fusion) (Lee et al., 2021). Our current study demonstrates that nuclear DNA damage that occurs spontaneously and increases with age is sufficient to drive all of the metabolic changes associated with retinal aging and degeneration.

Mitophagy is a quality control mechanism that enables the degradation of damaged mitochondria and restores cellular homeostasis in response to stress, and is critical for regulating mitochondrial quality and cellular homeostasis and function (Iorio et al., 2021). Lysosomal dysfunction impacts mitophagy, leading to elevated mitochondrial mass and, in turn, altered mitochondrial metabolism (Plotegher & Duchen, 2017). Irregular mitochondrial shape and size, lower ATP production, reduce mitochondrial membrane potential, impair mitochondrial fission/fusion balance, lower basal and maximal respiration, and decreased mitophagy is reported in aging RPE (Tong et al., 2022). Diminished RPE mitophagy along with reduced vision are observed in mice with low expression of proteins involved in mitochondrial biogenesis (PGC1α), antioxidant response (NFE2L2), or lysosomal acidification (CRYBA1) (Felszeghy et al., 2019; D. Sinha et al., 2016). The mitochondrial mass is elevated in *Ercc1* ^*−/Δ*^ mice, due to impaired mitophagy. Supporting this concept, the expression of proteins related to mitophagy (PINK1 and PARKIN) was significantly reduced in the *Ercc1* ^*−/Δ*^ mice and aged WT mice compared to healthy adult mice (Figure 6g). Notably. DNA damage and markers of cellular senescence were detected as early as 2‐months‐of‐age, but mitochondria/mitophagy defects were not, suggesting that mitochondrial dysfunction is downstream of cellular senescence.

To summarize, systemic deficiency of the DNA repair gene *Ercc1*, to drive the accelerated accumulation of spontaneous, endogenous DNA damage, is sufficient to cause a loss of vision and age‐related changes in the neural retina and RPE of the eye. DNA damage promotes senescence of neural retina, RPE cells, and organelle and metabolic changes that drive retinal degeneration. This suggests that the *Ercc1* ^*−/Δ*^ mice may be useful for additional mechanistic studies on age‐related retinal degeneration and rapidly testing therapeutic interventions. Our work also reinforces the notion that drugs targeting senescent cells (Niedernhofer & Robbins, 2018) or oxidative DNA damage (Robinson et al., 2018) may prove to be a promising therapeutic treatment against AMD.

## AUTHOR CONTRIBUTIONS

A. N. led this study. S. H. M. and W. W. H. conducted the E. R. G. studies. L. L. J. and L. K. M. did the O. K. R. experiments. H. R. did histology. D. S. K. and W. C. conducted fundus photography and blood vessel leakage experiments. T. K. H. and C. W. helped with qRT‐PCR and western blot. R. D. O., L. A., and M. J. Y. helped with animal husbandry, transport, and necropsies. A. N., S. H. M., L. L. J., H. R., M. J. Y., L. K. M., D. S. K. and L. J. N. helped with experimental design and data interpretation. A. N. wrote the manuscript. All authors contributed to editing.

## FUNDING INFORMATION

This work was supported by The University of Minnesota Foundation and a generous donation from Mrs. Cecilee A. Faster. AN, CW, RDO, LA, MJY, PDR and LJN were supported by NIH/NIA (R01AG063543, U19AG056278, and U54AG079754). Work in DSK laboratory is supported in part by an unrestricted grant from Research to Prevent Blindness to the Gavin Herbert Eye Institute at the University of California and by the NIH/NEI U01EY034594 and P30EY034070.

## CONFLICT OF INTEREST STATEMENT

None declared.

## Data Availability

The data that support the findings of this study are available on request from the corresponding author.
